# Monitoring of the Homogeneity of Primer Layers for Ink Jet Printing on Polyester Fabrics by Hyperspectral Imaging

**DOI:** 10.3390/polym16131909

**Published:** 2024-07-04

**Authors:** Olesya Daikos, Tom Scherzer

**Affiliations:** Leibniz Institute of Surface Engineering (IOM), Department of Materials Characterization and Analytics, Permoserstr. 15, D-04318 Leipzig, Germany; olesya.daikos@iom-leipzig.de

**Keywords:** hyperspectral imaging, primer layers, polyester fabric, application of coatings by ink jet printing, homogeneity of the coatings, application weight, in-line monitoring, partial least squares algorithm

## Abstract

Untreated polyester films and fibers can be hardly printed or coated, in particular if aqueous inks or lacquers have to be applied. Therefore, an adequate primer layer has to be applied first. A cationic polymer formulation based on poly(dimethylamine-co-epichlorohydrin-co-ethylenediamine) (PDEHED) was used as primer layer for digital printing on polyester fabrics. Because of the exceedingly high requirements on the homogeneity of such layers, hyperspectral imaging was used for qualitative and quantitative monitoring of the distribution of the primer layer on the textiles. Multivariate data analysis methods based on the PLS algorithm were applied for quantification of the NIR reflection spectra using gravimetry as a reference method. Optimization of the calibration method resulted in various models with prediction errors of about 1.2 g/m^2^. The prediction performance of the models was proven in external validations using independent samples. Moreover, a special ink jet printing technology was tested for application of the aqueous primer formulation itself. Since possible clogging of jet nozzles in the print head might lead to inhomogeneity in the coatings such as missing tracks, the potential of hyperspectral imaging to detect such defects was investigated. It was demonstrated that simulated missing tracks can be clearly detected. Consequently, hyperspectral imaging has been proven to be a powerful analytical tool for in-line monitoring of the quality of printability improvement layers and similar systems.

## 1. Introduction

Polyester textiles are mechanically robust, dimensionally stable, chemically and weathering-resistant, water-repellent and food safe. Therefore, they are used not only for clothing, in particular sports and outdoor clothing, but also in numerous technical applications such as home textiles, driving and conveyor belts, as reinforcement material in polymer and rubber hoses and belts (e.g., in food processing or for membranes), in printing technology (printing blankets, screen printing), for interior design in automotive engineering and similar means of transportation, in filter technology, for safety work clothing, for sails on yachts and in many other applications as well. The cost efficiency, mechanical stability, white color and weathering resistance of polyester fabrics makes them interesting for banner ads, flags and similar applications. Such products have to be printed with messages and complex images for sales promotion. However, the chemical inertness of polyester makes it rather difficult to print textiles, for example, by screen or ink jet printing. For this reason, a special primer coating or printability improvement layer has to be applied first to the textile fabric, which has to provide both excellent adhesion to the polyester fabric and outstanding printability. Both natural carbohydrates such as carob gum derivatives and synthetic polymers such as special cationic formulations have been used as primer layers on polyester fabrics.

The finishing of technical textiles with special functionalities such as flame retardancy, optical brightening, chemical or microbiological resistance, etc., or with adhesion promoters is typically achieved by aqueous impregnation in large converting plants, which may have a working width of several meters. Impregnation is a rather fail-safe and in general cost-efficient technology for textile finishing. However, it has the drawback that large amounts of the corresponding aqueous solution or dispersion have to be provided in order to fill the large trough of the padder even if only small amounts of textile (e.g., a few hundred meters) have to be finished. At the end of the process, the remaining finishing solution has to be pumped down and either stored if storable or disposed of, which is involved with costs.

Ink jet printing is used in textile converting not only for image printing with high resolution, as mentioned above, but also for the production of wall-to-wall carpets to be used in hotels, restaurants or offices. This kind of carpets may be provided with regular patterns, which are made by a special ink jet printing technology. The printers are suited for four-color printing. Hence, the printing heads are equipped with four tanks with a volume in the order of one liter. For economic reasons, it is therefore regarded to be useful to test the potential of this ink jet technology for finishing. The tanks may be filled with a conventional finishing solution of a flame retardant or a primer. Of course, it has to be made sure that the viscosity of the formulation is in the adequate range and that it does not contain any particles which might clog the printing nozzles. The advantage of this approach is the fact that it requires much less solution since only the small tank on the probe head has to be filled. So, it would be no longer necessary to provide large amounts of the finishing formulation if only little textile were to be treated.

Nevertheless, in spite of careful filtering of the formulation before printing, there remains some risk of clogging of jet nozzles in the printing head, e.g., due to agglomeration. This leads to untreated areas on the textile surface at which later printing by conventional printing inks fails. The clogging of single nozzles leads to tracks in the coating, whereas the clogging of several neighboring nozzles causes larger untreated areas. Missing tracks are particularly devastating since they are hardly to discover in the visible range but might extend to hundreds or even thousands of meters of converted textile web. However, both kinds of defects are fatal for printability improvement layers with respect to their application. For this reason, any kind of untreated area has to be detected reliably.

Hyperspectral imaging seems to be the perfect analytical method for this task. Due to the use of hyperspectral cameras, it combines high spatial resolution, which is required for the detection of missing tracks, with the outstanding potential of NIR reflection spectroscopy for process control. In the past, it has been used in numerous applications in quite different areas ranging from agriculture and food processing [1,2] via polymer processing [1,2] and production of pharmaceuticals [1,2,3] to materials identification in the sorting of plastic waste [4,5,6], glass [7] or asbestos containing construction materials [8]. Furthermore, more exotic applications are located in forensics [9,10], art conservation and authentication [11,12,13,14], archaeology [15,16], medicine and biotechnology [1,2,17] or even in geology [18].

Although one might assume that it suggests itself, very little work has been conducted so far to study 2D or even web-like materials such as paper, cardboard, textiles, coatings and similar systems by hyperspectral imaging. Regarding coatings, white pigmented UV-cured coatings were analyzed by this technique with respect to the acrylate conversion after irradiation as well as their thickness [19,20]. In the case of textiles, several studies have been carried out to develop methods for identification and classification of used textiles in analogy to plastic waste sorting [21,22,23,24]. Some further studies dealt with the use of hyperspectral imaging in textile converting, in particular in lamination [25], coating and finishing by impregnation [20,26,27,28,29,30] using either hyperspectral cameras or fiber-based NIR multiplex spectrometer systems [29]. In the case of finishing, mostly flame retardants or adhesion promoters were investigated. Both quantitative parameters such as application weight or residual moisture content after drying and more qualitative aspects such as homogeneity and freedom from surface defects were considered. It was proven that the sensitivity and the spatial resolution of hyperspectral imaging are sufficient for this kind of investigations on web-like materials.

In the present study, the spotlight is turned on thin printability improvement layers based on a cationic primer formulation, which is applied by ink jet printing as a technique that is unusual for such applications. The challenges of this specific problem consist in the rather low thickness of the layers, the high requirements concerning their homogeneity and in particular in the detection of possible missing tracks caused by the possible clogging of jet nozzles in the probe head of the printer.

## 2. Experimental

### 2.1. Materials and Sample Preparation

Printability improvement layers were prepared with a cationic polymer formulation based on poly(dimethylamine-co-epichlorohydrin-co-ethylenediamine) (PDEHED; Tubijet Sharp D 201; CHT Germany GmbH, Tübingen, Germany). The clear to pale yellow liquid has a solids content of 50 wt% and is miscible with water in any ratio.

Layers were applied to a white polyester fabric with a weight of 135 g/m^2^. Samples for calibration and validation as well as some samples with specific inhomogeneities were prepared by impregnation of the textile in solutions of the cationic primer with various concentrations in order to obtain samples with different applications weights. After drying, these application weights were determined by gravimetry using an analytical balance.

A few samples were printed by a special ink jet printing technology using a Chromojet Tabletop Printer (Zimmer Maschinenbau GmbH Digital Printing Systems, Kufstein, Austria). The primer solution was diluted to a concentration of 8% and filled into one of the tanks of the probe head. Only one jet module with eight 200 µm nozzles was used. The solution was printed at a speed of 0.3 m/s by application of a pressure of 3.5 bar. Different application weights were obtained by different pixel densities. The size of the printed area was 20 × 20 cm on a 30 × 30 cm textile sample.

### 2.2. Hyperspectral Imaging

Hyperspectral imaging was carried out with a pushbroom-type NIR camera (KUSTA1.9MSI, LLA Instruments GmbH, Berlin, Germany), which is based on a grating spectrograph and a Peltier-cooled InGaAs photodiode detector with 192 × 96 pixels (spatial × spectral resolution), which covers a range from 1320 to 1900 nm. The camera is mounted above a conveyor with a belt width of 500 mm (Axmann Fördersysteme GmbH, Zwenkau, Germany). The belt is coated with black polyurethane with a matted surface. The resulting low reflectance prevents a significant disturbing effect of the belt on the spectra of the samples lying on it. In order to adapt the field of view of the NIR camera to the width of the belt, its standard objective (designed for a width of 1 m) was replaced by a F2.0/15 mm objective (Specim, Oulu, Finland). Further fine adjustment of the vertical position of the camera above the conveyor was carried out in order to make sure that exactly the full width of the belt is projected to the camera detector. Considering the width of the belt and the number of pixels available for spatial resolution, a lateral resolution of 2.6 mm per pixel was achieved.

Samples on the belt need to be illuminated uniformly and with adequate luminance in the relevant near-infrared range. The used camera system was equipped with an illumination unit consisting of two lamp modules (PMAmsi500 LR30; LLA Instruments GmbH) mounted on both sides of the field of view of the objective. Each module was equipped with four 120 W halogen lamps (Haloline Eco, Osram, Munich, Germany). A roof-shaped arrangement of the modules (i.e., with a tilt against each other) minimizes specular reflections from the sample on the belt to the camera sensor. A scheme of the experimental setup can be found in ref. [25].

Hyperspectral imaging was carried out at a line speed of 10 m/min, which corresponds to that of most textile finishing processes. During passage below the camera, reflection spectra of the samples were collected across the complete surface of the samples.

### 2.3. Multivariate Data Analysis

Chemometric approaches [31,32,33] were applied for the extraction of quantitative information from the recorded spectral data. Regression of the data was realized by application of the partial least squares (PLS) algorithm using gravimetric data of the application weight as reference values. Various spectral pretreatments as well as variations of the spectral range under consideration were applied to the spectra (either individually or in combination) to optimize the calibration models. The determination of the optimum number of eigenvectors (i.e., latent variables) of each model was carried out using the test set method, which requires an independent validation set of samples. The standard error of prediction (SEP), the root mean square errors of calibration and prediction (RMSEC, RMSEP), the bias and the coefficient of determination (R^2^) were calculated in order to enable a comparison and rating of the various PLS models. Generally, the model with the lowest RMSEP and the highest R^2^ is selected for quantitative analysis of the in-line spectra and generation of quantitative spectral images from the corresponding hypercube.

Multivariate data analysis was executed with the Kusta software package (version 19.7.0) provided with the camera. Chemometric modelling was carried out with the KustaSpec module, whereas hyperspectral images were obtained with the KustaBelt module using the developed models. Further details about specific data pretreatments, PLS modelling and the resulting spectral images of the various samples are given in the Results and Discussion section.

## 3. Results and Discussion

### 3.1. Calibration and Validation

Printability improvement layers serve as primers for conventional printing inks on textiles such as polyester fabrics, which are otherwise hardly printable without this kind of pretreatment because of the poor adhesion of such inks on this polymer. Cationic primers such as PDEHED are typically applied by impregnation in an aqueous solution but might be applied by other techniques such as ink jet printing as well if the viscosity of the formulation fits to the specific requirements of these processes. It was the intention of this work not only to monitor the homogeneity of the resulting primer layers on the polyester fabric, which is a basic requirement for flawless printing, but also to estimate the amount of primer applied to the textile. Quantitative monitoring by NIR methods requires precise calibration of the spectra to the parameter of interest as very first step. Calibration samples needed for this process have to be carefully characterized, since this significantly determines the precision of the later predictions. For the determination of the application weight, characterization is performed by gravimetry. In the case of ink jet printing, it is rather difficult to apply the ink to the total surface of the substrate; that means unprinted margins surround the printed area. However, these margins prevent precise gravimetric determination of the application weight. For this reason, calibration and validation samples used in the present study were prepared by impregnation, which inherently ensures finishing of the complete area of the textile samples. Variation of the application weight was achieved by stepwise dilution of the finishing solution. Subsequently, samples were dried at room temperature. After gravimetric analysis, the samples were split into three sets for calibration as well as internal and external validation. During this procedure, it was made sure that all three sets cover the complete range of application weights.

**Figure 1:**
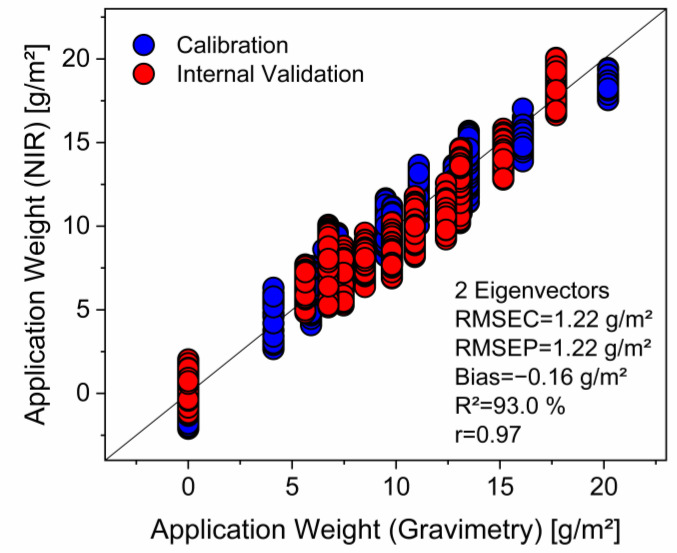
PLS calibration model for the application weight of the cationic PDEHED polymer on polyester fabric.

For all three sets, images were recorded with the hyperspectral camera. NIR reflection spectra extracted from an area in the center of each sample (about 1000 spectra per sample) were used for the calibration process (or later for external validation). Finally, a calibration model was built up from these data. In order to optimize the model (i.e., to reduce the number of eigenvectors and to minimize the prediction error), various spectral pretreatments were applied to the data set. Moreover, the spectral range that is considered by the model was varied as well. For each model, a number of specific statistical parameters (RMSEC, RMSEP, SEP, bias, R^2^) were calculated. A selection of some of the developed calibration models is listed in Table 1. It was stated above that in general, the model with the lowest RMSEP and the highest R^2^ is selected as optimum model. In the present study, it is obvious that at least four models in Table 1 (1, 2, 4 and 5) have very similar values in this regard. However, the second and the fifth model are based on three eigenvectors, whereas the other two require only two latent variables. Moreover, normalization is regarded to have the lowest impact on the spectra, even though baseline correction is also a rather soft method, for example, in comparison to differentiation. It is well known that the number of eigenvectors should be as low as possible with regard to the prediction stability of the model, and analogously this also applies to the extent of the pretreatment of the spectra. Consequently, the first model was selected for the conversion of the raw data of the images into quantitative images as well as for in-line analysis of impregnated and printed samples. Figure 1 shows the PLS regression of this model and includes data of both the calibration and the internal validation set.

**Figure 2:**
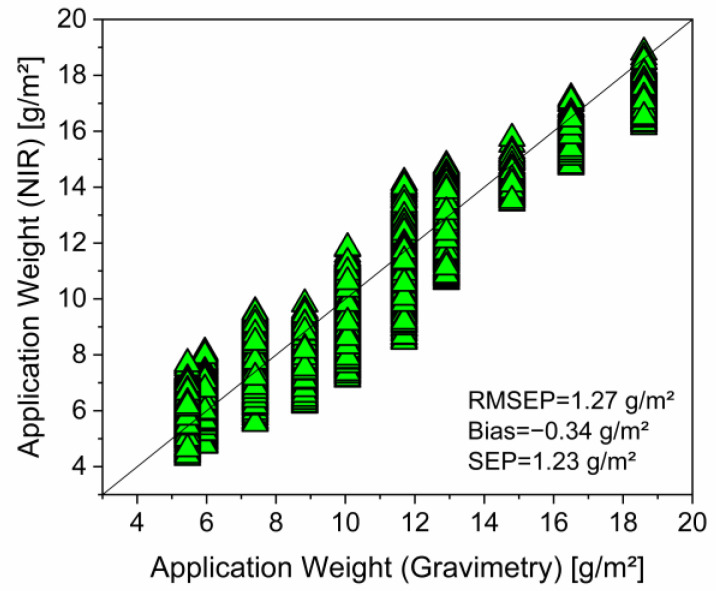
External validation of the calibration model shown in Figure 1 by prediction of the application weight of independent samples of the cationic primer on polyester fabric.

In the next step, the external validation, the prediction performance of this model was tested by its application to the third data set. The data of this set were not used in the calibration process. Consequently, the samples of this set can be considered as independent as it is required for a statistically sound model evaluation. Application weights of PDEHED on the polyester fabric were predicted from their hyperspectral images using the above calibration model and compared with reference values determined by gravimetry. Results are summarized in Figure 2.

Generally, the prediction error RMSEP in the external validation cannot be lower than the corresponding value in the calibration. However, if the first value is only a little higher than the second one, this indicates a high precision of the predicted data. The comparison with the reference data clearly confirms this statement. The bias has a rather low value as well, which indicates that systematic errors are largely insignificant. Consequently, the external evaluation proves the high prediction performance of the selected calibration model. Hence, it may be used for predictions in subsequent practical applications.

**Figure 3:**
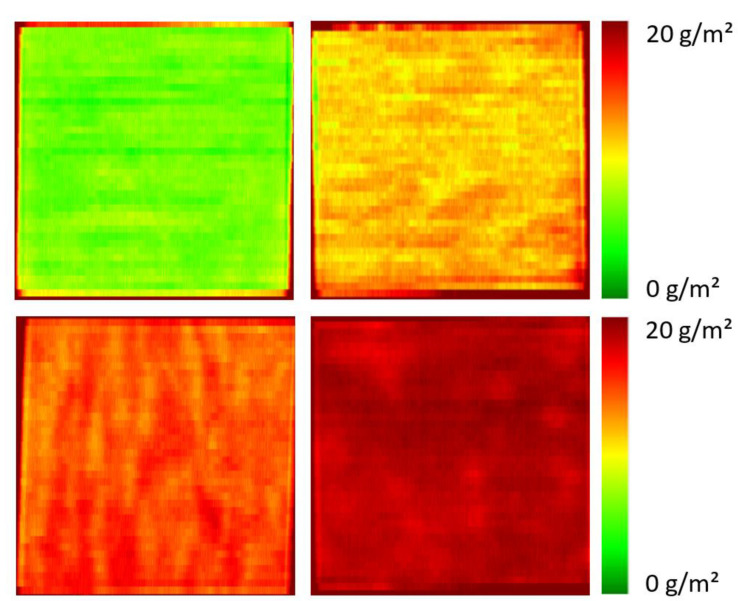
Spectral images of selected samples from the validation set. The gravimetric application weights of the cationic polymer are 5.9, 10.1, 12.9, and 18.6 g/m^2^, respectively (from **top left** to **bottom right**).

Figure 3 shows the quantitative spectral images of some samples from the third sample set. All images confirm a very high homogeneity of the primer layers on the polyester fabric as well as significant differences in the amount of PDEHED applied to the textiles.

### 3.2. Detection of Thickness Variations and Inhomogeneity

**Figure 4:**
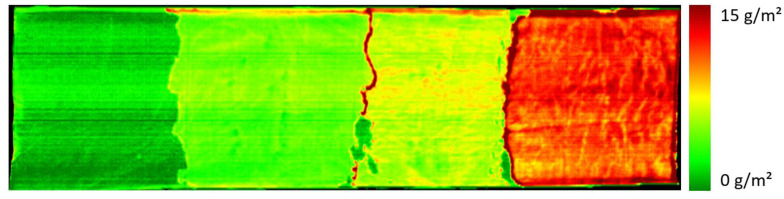
Spectral image of a textile web impregnated with solutions of PDEHED with different concentrations (4, 8 or 16% / 2nd to 4th area from **left** to **right**). The leftmost area was untreated (substrate only). The averaged predicted application weights were 0, 4.4, 6.5 and 11.3 g/m^2^, respectively.

In order to further evaluate the suitability of the developed calibration model for the prediction of the surface density of the PDEHED formulation on polyester fabric, we prepared a textile web with several consecutive areas with different application weights, which was achieved by successive impregnation in solutions with different concentrations of the cationic polymer. After each impregnation step, the textile web was dried before further treatment. The spectral image of the resulting sample is shown in Figure 4. The total length of the textile web was 80 cm.

It is evident that the differences in the application weight can be clearly seen in the image. The irregular transitions from one zone to the next result from the specific sample preparation process. It is obvious that gravimetry cannot be executed for this kind of sample, so a comparison with such data is not possible. For each zone, an average application weight was determined from the predicted data using the complete homogeneous part of that zone and averaging the predicted values. These averaged values are given in the figure legend. They correlate quite well with the concentrations of the solutions used for the preparation of each zone.

**Figure 5:**
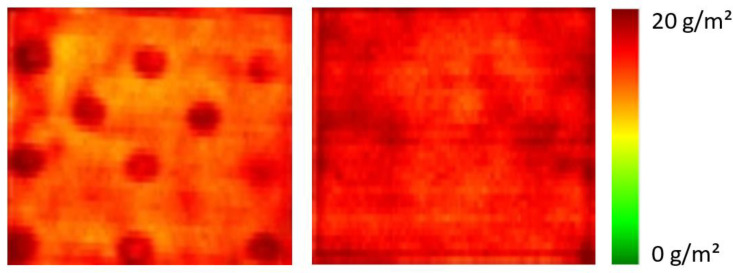
Spectral images of polyester fabrics finished with PDEHED and then dried in a drying oven at 100 °C. The sample on the **left** side was directly placed on the perforated tray, whereas the **right** was placed on a glass plate.

Typically, samples prepared by impregnation were dried at room temperature simply by storage in the lab. However, some samples were dried in a drying oven at a temperature of 100 °C. An example is shown in Figure 5. The left spectral image shows a pattern of circular structures. The circles result from the perforated tray in the drying oven. Due to the convection in the oven, the textile dried faster in the area of the holes than in their surroundings. This probably led to diffusion of the finishing solution from the surrounding to the area above the hole and consequently to an accumulation of the cationic polymer in this area. Hyperspectral imaging is sensitive enough to detect this effect. It shows not only the higher concentration in the hole areas, but also a corresponding decrease in the application weights around most of the holes. If the samples are placed on glass plates during drying in the oven, the convection effect is avoided as can be seen in the image on the right side of Figure 5. The distribution of the cationic polymer on the polyester fabric is largely homogeneous.

### 3.3. Analysis of Textile Samples Finished by Ink Jet Printing

**Figure 6:**
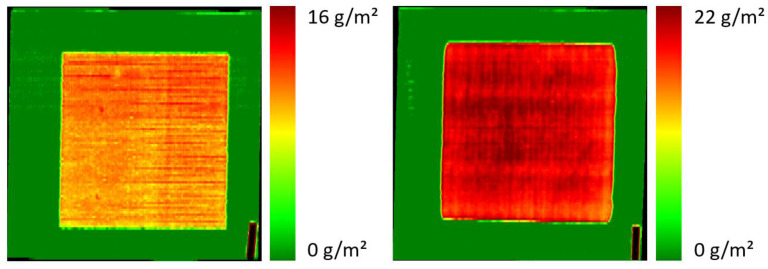
Spectral images of polyester fabrics finished with PDEHED by ink jet printing. The relative pixel densities were 10% (**left**) and 20% (**right**).

All samples used for calibration, validation or the detection of nonuniformities so far were prepared by impregnation in aqueous solutions of the PDEHED formulation. As mentioned above, it was the final objective of this work to develop an analytical method to monitor the finishing of textiles by ink jet printing. Hence, a number of samples were prepared by this new technique. A diluted solution was printed to the polyester textile at a rather low speed of 0.3 m/s. On the one hand, the concentration of the solution has to be low enough to avoid clogging of the jet nozzles in the printing head, but on the other hand, an excessively strong dilution leads to excessive spreading of the applied solution in the textile. Finally, a solution with a solids content 8 wt% was found to be the optimum recipe. Figure 6 shows two examples of squares with an edge length of 20 cm that were printed to the polyester fabric. It is obvious that the squares have very sharp edges, indicating that spreading of the solution in the textile did not occur.

Using hyperspectral imaging and the calibration model in Figure 1, the application weights in the printed areas of the two samples were predicted to be 9.9 and 19.8 g/m^2^, respectively. It is evident that the second value is exactly twice as high as the first one, which perfectly correlates with the pixel densities. This finding may be regarded as a further confirmation of the performance of the developed calibration model. Obviously, gravimetric application weights cannot be given for comparison since only parts of the textile surface are printed. However, close correlation between the spectroscopic method and gravimetry has been shown above. Thus, hyperspectral imaging could be used for continuous quantitative control of the coating weight applied by ink jet printing.

**Figure 7:**
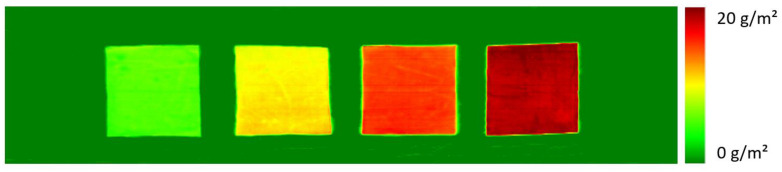
Monitoring of the local finishing of a polyester fabric with PDEHED by ink jet printing. The relative pixel densities used for the various squares were 2, 5, 10 and 20% from left to right. The width of the textile web was 40 cm. Spectral imaging was carried out at a line speed of 10 m/min.

In order to simulate continuous monitoring in process control, sequences of consecutive squares were printed to polyester webs. Each square was printed with another pixel density, but with the same concentration of the PDEHED solution. Afterwards, the finished textiles were imaged by the hyperspectral camera. An example is shown in Figure 7. The different application weights resulting from the various pixel densities are clearly apparent from the image. Quantitative values determined from the spectral images (in the same way as described above) were 2.3, 4.8, 10.2 and 20.1 g/m^2^, respectively, which again correlates quite well with the corresponding pixel densities. Moreover, the homogeneity of the application weight within each square is rather high, which emphasizes the outstanding potential of the specific application technology in this regard compared to impregnation. Only minor diffusion is observed between printed and untreated areas, leading to rather sharp edges of the squares even in this semi-continuous printing process.

**Figure 8:**
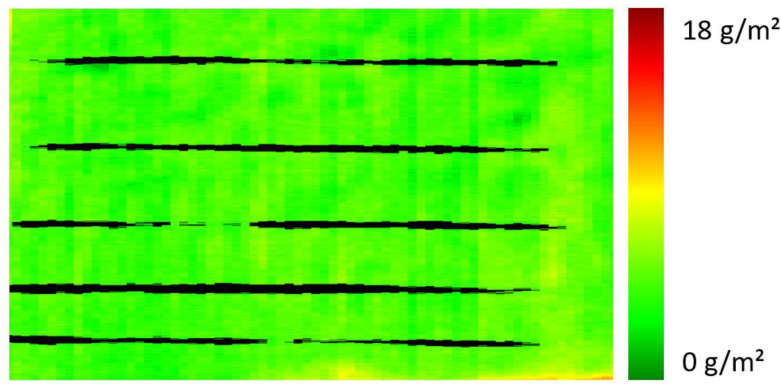
Spectral image of a finished polyester fabric with simulated clogging of several jet nozzles in the printing head.

Using ink jet printing technology may imply the risk of clogging of the jet nozzles in the printing head. The clogging of single nozzles might lead to missing tracks in the coating (which are extremely difficult to detect visually), whereas the clogging of several neighboring nozzles might cause larger inhomogeneity in the finish. Since this would be destructive for printability improvement layers with respect to their application, such flaws have to be detected reliably. The potential of hyperspectral imaging to discover missing tracks was tested by purpose-made samples with such defects. An example is shown in Figure 8: the applied printability improvement layer based on PDEHED is damaged by a number of simulated tracks without primer, which will later fail to take printing inks. It is clearly apparent that hyperspectral imaging is able to detect the missing tracks, which allows stopping the production process to clean the probe head and to eliminate the deficient section of the web from the production output. The irregular width of the simulated tracks in Figure 8 as well some discontinuities are related to the manual preparation process.

## 4. Conclusions

It was the intention of this paper to develop an analytical method based on hyperspectral imaging for qualitative and quantitative monitoring of the preparation of printability improvement layers on polyester fabrics, which is suitable for in-line process control. In particular, a cationic polymer formulation based on poly(dimethylamine-co-epichlorohydrin-co-ethylenediamine) (PDEHED) was used as primer on the textile. Multivariate data analysis methods based on the PLS algorithm were applied for quantification of the NIR reflection spectra using gravimetry as the reference method for calibration. Optimization of the calibration method resulted in various models based on two or three eigenvectors and with prediction errors of about 1.2 g/m^2^. Their prediction performance was confirmed in external validations using independent samples. Moreover, it was shown that the model that was selected finally was able to detect various thicknesses of the applied PDEHED layers as well as deviations in homogeneity.

Usually, finished textiles are prepared by impregnation. Special ink jet printing technologies may have potential for cost-efficient textile finishing, since they require much less finishing solution or dispersion. However, possible clogging of jet nozzles in the printing head might lead to inhomogeneity in the coating such as missing tracks. Since this would be destructive for printability improvement layers with respect to their application, hyperspectral imaging was used to monitor the quality of layers applied by ink jet printing. It was proven that the application weight closely correlates with pixel density. Moreover, it was demonstrated that simulated missing tracks resulting from clogged nozzles can be clearly detected by this method. Consequently, it can be concluded that hyperspectral imaging is a very powerful tool for in-line monitoring of the quality of printability improvement layers and similar systems.

## Figures and Tables

**Table 1:** Parameters of various PLS models based on different spectral pretreatments of the spectra and/or spectral ranges considered. The model selected for further analysis is highlighted.

Spectral Range [nm]	Preprocessing	Eigenvectors	RMSEC [g/m^2^]	RMSEP [g/m^2^]	R^2^ [%]
*1325–1600*	*Normalization*	*2*	*1.22*	*1.22*	*93.0*
1325–1900	Normalization	3	1.23	1.25	92.7
1600–1900	Normalization	4	2.27	2.47	73.5
1325–1600	Baseline Correction	2	1.19	1.24	93.0
1325–1900	Baseline Correction	3	1.21	1.30	92.6
1600–1900	Baseline Correction	4	2.48	2.58	70.0

## Data Availability

All data are present inside the graphs. No additional data will be put online, further inquiries can be directed to the corresponding author.
